# Voluntary Counseling and Testing for HIV in Rural Area of Democratic Republic of the Congo: Knowledge, Attitude, and Practice Survey among Service Users

**DOI:** 10.1155/2015/281093

**Published:** 2015-08-09

**Authors:** Mida Kautako-Kiambi, Mathilde B. Ekila, Smith Kama-Lemba, Roger Wumba, Michel N. Aloni

**Affiliations:** ^1^Centre de Santé de Référence et Maternité de la Cité de Mbanza-Ngungu, Mbanza-Ngungu, Bas-Congo, Democratic Republic of the Congo; ^2^Division of Infectious Diseases, Department of Internal Medicine, University Hospital of Kinshasa, School of Medicine, University of Kinshasa, Kinshasa, Democratic Republic of the Congo; ^3^Department of Tropical Medicine, University Hospital of Kinshasa, Faculty of Medicine, University of Kinshasa, Kinshasa, Democratic Republic of the Congo; ^4^Division of Hemato-Oncology and Nephrology, Department of Pediatrics, University Hospital of Kinshasa, School of Medicine, University of Kinshasa, Kinshasa, Democratic Republic of the Congo

## Abstract

*Aims.* To determine the prevalence of HIV, the level of sexual risk for HIV, and determinants of VCT attendance among adult population living in a rural area.* Methods.* A cross-sectional study was conducted in Mbanza-Ngungu, Democratic Republic of the Congo. An anonymous questionnaire was designed to extract relevant data.* Results.* In our cohort, 69% were respondents of more than 24 years of age and the single marital status was most represented (64.1%). A high proportion of respondents (90.6%) visited VCT service for requiring information (good acceptability). Positive test for HIV was reported in 9.4% of respondents. In this cohort, 49.6% of respondents had declared themselves to never use condom. In binary analysis, there was association between positive HIV test and age (*p* = 0.04) and religions (*p* = 0.02). In this cohort, it was observed that positive HIV test was significantly associated with confidentiality (*p* = 0.02). However, there was no association between positive HIV test and condom use (*p* = 0.25), knowledge of VCT (*p* = 0.81), service requested (*p* = 0.20), and previous HIV test (*p* = 0.68).* Conclusions.* Preventive information for AIDS should be recommended in the population living in rural zone.

## 1. Introduction

Since 80s, HIV/AIDS infection remains one of the most devastating diseases worldwide. Approximately 33 million people are living with HIV and among them 97% are living in developing countries. Developing countries account for 97% of the HIV cases reported in the world. Sub-Saharan Africa is the first region of the world affected by the disease [1].

PNUD-DRC in 2009 report states that the prevalence of AIDS is around 4% and approximately 1,034,086 infected people (more than 15 years of age) are affected by the disease in the Democratic Republic of the Congo (DRC) [2]. It is be estimated that 80% of people who tested positive for HIV were in later stages and many infected individuals ignore their HIV status [3].

The situation requires measures to be strengthened, including public Voluntary Counseling and Testing (VCT) which is an entry point for prevention, education, care, and support for HIV infected individuals [4, 5]. However, rare published studies focused on persons who access these services in this country [6].

We therefore conducted a cross-sectional study in Mbanza-Ngungu, a semirural area located in the western part of the DRC. The objectives of this study were to determine the prevalence of HIV, the level of sexual risk for HIV, and determinants of VCT attendance among adult population living in this area. This piece of information will give the whole view of patients and may serve to rule out general public health in our midst.

## 2. Materials and Methods

### 2.1. Ethical Approval

Pending the installation of an ethical committee in the province of Bas-Congo of the DRC and the central health zone of Mbanza-Ngungu hospitals, the study was assessed and approved by the Provincial Health Officer (the highest ranked medical authority in the Oriental Province) and the Director's Board of Nsona-Nkulu Hospital and Maternity of Mbanza-Ngungu city. The aim and the procedures of the study were explained to the respondents. A written informed consent signed by participants was obtained before their inclusion. The respondents were informed that they could withdraw anytime without further obligation. Confidentiality of data was maintained.

### 2.2. Study Design

This cross-sectional study was conducted between January 2011 and July 2011 in Mbanza-Ngungu, Democratic Republic of the Congo. In this area, the HIV prevalence was estimated to be around 1.1% in the adult population between 15 and 49 years of age and from 3.3% to 4.9% for pregnant women [2]. Mbanza-Ngungu is a semirural territory in Bas-Congo Province in the western part of the Democratic Republic of the Congo; it has a population of nearly 100,000 people.

### 2.3. Study Population

The study included all individuals who visited the VCT services. Pregnant women were excluded from the study. The responders were classified in two categories of age: group 1 (≤24 years of age) and group 2 (>24 years of age).

This study was conducted in two VCT services located in health facilities in Mbanza-Ngungu, namely, Nsona-Nkulu hospital and Maternity of Mbanza-Ngungu city. They were selected for the study because they offered information, education, and management for the population of the city.

### 2.4. Data Collection

The respondents were selected and included in this study using a simple random sampling method. An anonymous questionnaire was designed to extract relevant data from the case records. The questionnaire was prepared in French and translated in Kikongo (the predominant language in Mbanza-Ngungu). The questionnaire was pretested in 20 respondents not selected for the study.

The respondents selected for the study had completed an anonymous questionnaire. The structured questionnaire is self-administered. The structured questionnaire contained of a total of questions addressing three axes: (i) demographic data (age, gender); (ii) prevention behavior for HIV/AIDS; (iii) knowledge and attitude.

### 2.5. Data Analysis

Data from the returned questionnaires were stored confidentially and were manually entered into a microcomputer using Microsoft Office Excel 2010. After data cleaning (control for quality and coherence), they were exported on SPSS 19.0 for statistical analysis. Descriptive analyses were used to get mean for quantitative variables and proportion for all the qualitative variables. The confidence interval at 95% was calculated. Chi2 tests, Fisher's exact tests, and Student's *t*-tests were used for comparisons between variables. For identification of factors associated with the use of VCT services, we performed a binary logistics analysis. Differences were considered significant when *p* < 0.05.

## 3. Results

### 3.1. Sample Characteristics

A total of 234 respondents gave informed consent from 247 received during the period of the study. The participation rate was 95%. These data were used for analysis.

Sociodemographics and general characteristics of the respondents are presented in Table 1. The proportion of males and females visitors was similar. In our cohort, 161 (69%) were respondents more than 24 years of age (Table 1). Only 4% of respondents achieved the tertiary education level. Regarding the occupation, 53% of the respondents were categorized as employed. The respondents with single marital status were most represented (64.1%). The majority (77.8%) of the respondents were Christians. Other details are reported in Table 1.

### 3.2. Knowledge and Attitude

Knowledge and attitude of respondents are summarized in Table 2. Forty-seven percent of the respondents know VCT service. The three main sources of VCT information are health agents, the vicinity, and the media. Other details are reported in Table 2.

The most common reasons of the visits were the curiosity, HIV retesting, and the context of another disease. Other reasons are summarized in Table 2. Only 8.4% of the visits were due to counseling before marriage or a new relationship. A high proportion of respondents (90.6%) visit VCT service for requiring information (*good acceptability*). Two percent of respondents had supposed that they were infected and expected a positive HIV test. Positive test for HIV was reported in 22 (9.4%) respondents and 13 (5.6%) had an undetermined result. A total of 134 (57.3%) respondents were screened for HIV infection in the past. A minority (0.8%) of them had confirmed using condom during each suspected sexual relation. In contrast, 116 respondents (49.6%) had declared themselves to never use condom. Among them 65 (56%) were single. Forty-seven percent of responders agree to inform their sexual partner if the HIV test was positive. Other details are shown in Table 2.

Frequencies of positive test for HIV in 234 responders according sociodemographic characteristics are summarized in Table 3.

### 3.3. Binary Analysis

The results of the bivariate analysis between positive HIV testing and sociodemographic characteristics of respondents are shown in Table 4. There was no association between positive result for HIV test and gender (*p* = 0.47), education level (*p* = 0.64), job category (*p* = 0.93), and marital status (*p* = 0.98). However, there was association between positive HIV test and age (*p* = 0.04) and religions (*p* = 0.02).

Table 5 shows the association between positive HIV test and behaviors and attitude of the study population. In this cohort, it was observed that positive HIV test was significantly associated with confidentiality (*p* = 0.02). However, there was no association between positive HIV test and condom use (*p* = 0.25), knowledge of VCT (*p* = 0.81), service requested (*p* = 0.20), and previous HIV test (*p* = 0.68).

## 4. Discussion

To our knowledge, the present study is one of the first attempts to describe characteristics and attitude about HIV/AIDS in individuals attending VCT in a rural area of Central Africa. VCT serves as an entry point and represents one of the strategies adopted by the international community to control HIV explosion [4].

In this study, the HIV prevalence was 9.4%. This trend is higher than those observed in previous studies in another part of the province of Bas-Congo where the prevalence varies between 1.1% and 5.7% [7]. This difference can be explained by the target population in this study. This prevalence is probably increased in some high-risk specific groups attending the VCTs.

In Mbanza-Ngungu, the greater proportion of respondents with increased risk of HIV infection was individuals aged more than 24 years. Different studies reported similar results in the DRC [3, 8–10].

Educational level was not found to be a significant factor affecting the result of HIV test. Previous studies show that education level is a major determinant for HIV testing and counseling [9, 11, 12]. This situation could be explained by the underrepresentation of responders with high level education in a rural area in the context of the DRC.

In this cohort, job category was not found as a significant risk factor for positive HIV test. This result is not in accordance with those published in 2011 by the multisectorial program against HIV/AIDS of the DRC. This situation is probably due to underrepresentation of group with high risk of HIV infection (sex professionals, truck drivers, and female soldiers) in our cohort [13–15].

Christianity is found to be a significantly protective factor against HIV infection, in our cohort. A recent study in Tanzania found that religious beliefs have a significant influence on HIV-related stigma [16]. In Congolese rural area, Christianity coexists with several African traditional religions and sociocultural beliefs. By their convictions, African traditional religions could constitute a barrier for HIV disclosure and an obstacle for prevention strategies. However, recent studies in other provinces of DRC showed that Catholic (relative to non-Catholic Christian) religious affiliation was associated with an increased risk of HIV [10].

In our cohort, the positive HIV test was predominant in females. The female preponderance noted in this cohort was comparable with previous studies in Africa [3, 17].

In our cohort, there was no association between positive HIV test and marital status. This observation has been confirmed by subsequent studies [10]. However, in rural area in Kenya, married women were 4.8 times more likely to be HIV positive than those never married [18]. Similar observation was recently reported in Mozambique [19]. Efforts need to be strengthened to make couples aware of HIV spread and disclosure.

This study reveals that respondents with single marital status visited more VCT service than the couples. The low frequency of responders in couple might be related to the false idea that marital partnerships prevent from HIV in African culture [20, 21].

Our results show that respondents that are willing to disclose their status to a confidant had probably a negative HIV test. This suggests that they knew they are in good health and more of them (69%) have HIV testing in the past. It is important to note that 16% of respondents did not wish to disclose their HIV status and 51% of them have a sexual partner. These findings are comparable with previous studies in Africa [22, 23].

Sex with occasional partners was considered as risky sexual behaviors. The use of condom remains an effective preventing factor of HIV sexual transmission [24, 25]. In our cohort, only 0.8% of respondents have declared regularly using condom and approximately 50% of them use condom occasionally. Similar observations have been previously reported in other categories of population living in the DRC [26–28].

## 5. Conclusion

VCT is an entry point for HIV prevention and therapeutic support. This study shows the need to strengthen information on AIDS prevention in rural areas in the DRC. Preventive information on AIDS should be recommended in the population living in rural zone, an area at high risk, to contract HIV/AIDS. However, HIV/AIDS control requires a comprehensive approach, including the leaders and community education.

## Figures and Tables

**Table 1:** Sociodemographics and characteristics of the 234 study participants.

Variables	Frequency	Percent
Gender		
Male	117	50
Female	117	50
Age (years)		
≤24	73	31.2
>24	161	68.8
Education		
Primary	141	60.3
Secondary	84	35.9
Tertiary	9	3.8
Occupation		
Unemployed	55	23.5
Employed	124	53.0
Students	55	23.5
Marital status		
Single/widowed/divorced/separated	150	64.1
Married/cohabitation	84	35.9
Religion		
Christianity	182	77.8
Kimbanguism	14	6.0
Islam	5	2.1
Without religion	17	8.3
Animist	16	6.8

**Table 2:** Knowledge, attitude, and behavioral variables frequency (%).

Variables	Frequency (%)
Source of information	
Media (TV, radio, journal, etc.)	28 (12)
Health service, health care worker	110 (47)
Vicinity (sexual partner, family, friend, etc.)	68 (29.1)
Church	5 (2.1)
Other	23 (9.8)
Purpose of visit	
New sexual partner or marriage	22 (8.4)
Simple curiosity	94 (40.2)
Risk sexual relation	7 (3)
Sickness	31 (13.2)
Death from HIV in the family	11 (4.7)
Being oriented by VCT client or health agent	9 (3.8)
Rape	6 (2.6)
Retesting (after a first test)	41 (17.5)
Other	13 (5.7)
Service requested during visit	
Information	22 (9.4)
VCT	10 (4.3)
Information and VCT	202 (86.3)
HIV testing expected result by subjects	
Negative	112 (48.1)
Positive	5 (2.1)
Do not know	116 (49.8)
HIV testing result	
Negative	199 (85)
Positive	22 (9.4)
Undetermined	13 (5.6)
Previous HIV testing	
No	100 (42.7)
Yes	134 (57.3)
Sexual relation 3 months before VCT	
Yes	128 (54.7)
No	106 (45.3)
Use of condom	
Never	116 (49.6)
Sometimes	116 (49.6)
Always	2 (0.8)
Confidence of HIV test (if positive) with	
No body	37 (15.8)
Sexual partner	110 (47)
A family member	47 (20.1)
Pastor/priest	4 (1.7)
Other	36 (15.4)

VCT: Voluntary Counseling and Testing.

**Table 3:** HIV positive tests in 234 study participants according sociodemographics and characteristics.

Variables	Frequency	Percent
Gender		
Male	10/117	8.6
Female	12/117	10.3
Age (years)		
≤24	2/73	2.7
>24	20/161	12.4
Education		
Primary	14/141	9.9
Secondary	7/84	8.3
Tertiary	1/9	11.1
Job category		
Unemployed	6/55	10.9
Employed	15/121	12.4
Students	1/55	1.8
Marital status		
Single/widowed/divorced/separated	13/150	8.7
Married/cohabitation	9/84	10.7
Religion		
Christians	13/182	71.4
Kimbanguism	1/14	7.1
Muslim	1/5	20.0
Without religion	1/17	5.9
Animist	6/16	37.5

**Table 4:** HIV testing result from VCT versus sociodemographic variables.

Variables	Coefficient	*p* value	OR [95% CI]
Age	1.65	0.04	5.2 [1.1–24.7]
Education level	0.23	0.64	1.3 [0.5–3.3]
Job category	0.06	0.93	1.0 [0.3–4.1]
Religion	−1.27	0.02	0.3 [0.1–0.8]
Gender	−0.34	0.47	0.7 [0.3–1.8]
Marital status	0.01	0.98	1.0 [0.4–2.8]

VCT: Voluntary Counseling and Testing.

**Table 5:** HIV testing result from VCT versus attitude and behavioral variables.

Variables	Coefficient	*p* value	OR [95% CI]
Condom use	−0.56	0.25	0.6 [0.2–1.5]
Confidentiality	−1.1	0.02	0.3 [0.1–0.8]
VCT service knowledge	−0.12	0.81	0.9 [0.3–2.2]
Service requested	1.38	0.20	4.0 [0.5–33.6]
Previous HIV test	0.20	0.68	1.2 [0.5–3.1]

VCT: Voluntary Counseling and Testing.
